# Overcoming the Subject-Object Dichotomy in Urban Modeling: Axial Maps as Geometric Representations of Affordances in the Built Environment

**DOI:** 10.3389/fpsyg.2018.00449

**Published:** 2018-04-20

**Authors:** Lars Marcus

**Affiliations:** Spatial Morphology Group, Architecture and Civil Engineering, Chalmers University of Technology, Gothenburg, Sweden

**Keywords:** space syntax, affordances, urban modeling, sustainable urban design, spatial cognition

## Abstract

The world is witnessing unprecedented urbanization, bringing extreme challenges to contemporary practices in urban planning and design. This calls for improved urban models that can generate new knowledge and enhance practical skill. Importantly, any urban model embodies a conception of the relation between humans and the physical environment. In urban modeling this is typically conceived of as a relation between human subjects and an environmental object, thereby reproducing a humans-environment dichotomy. Alternative modeling traditions, such as space syntax that originates in architecture rather than geography, have tried to overcome this dichotomy. Central in this effort is the development of new representations of urban space, such as in the case of space syntax, the axial map. This form of representation aims to integrate both human behavior and the physical environment into one and the same description. Interestingly, models based on these representations have proved to better capture pedestrian movement than regular models. Pedestrian movement, as well as other kinds of human flows in urban space, is essential for urban modeling, since increasingly flows of this kind are understood as the driver in urban processes. Critical for a full understanding of space syntax modeling is the ontology of its' representations, such as the axial map. Space syntax theory here often refers to James Gibson's “Theory of affordances,” where the concept of affordances, in a manner similar to axial maps, aims to bridge the subject-object dichotomy by neither constituting physical properties of the environment or human behavior, but rather what emerges in the meeting between the two. In extension of this, the axial map can be interpreted as a representation of how the physical form of the environment affords human accessibility and visibility in urban space. This paper presents a close examination of the form of representations developed in space syntax methodology, in particular in the light of Gibson's “theory of affordances.“ The overarching aim is to contribute to a theoretical framework for urban models based on affordances, which may support the overcoming of the subject-object dichotomy in such models, here deemed essential for a greater social-ecological sustainability of cities.

## Introduction: the humans-environment relation in urban modeling

The world is witnessing unprecedented urbanization (United Nations, 2014), bringing extreme challenges to contemporary practices in urban planning and design. This calls for improved urban models that can generate new knowledge and enhance practical skill. Importantly, any urban model embodies a conception of the relation between humans and the physical environment. In urban modeling this is typically conceived of as a relation between human subjects and an environmental object (Wilson, 2000), thereby reproducing a humans-environment dichotomy.

Alternative modeling traditions, such as *space syntax* (Hillier and Hanson, 1984) that originates in architecture rather than geography, have tried to overcome this dichotomy. Central in this effort is the development of new representations of urban space, such as in the case of space syntax, the *axial map*^1^ (Hillier and Hanson, 1984). This form of representation aims to integrate both human behavior and the physical environment into one and the same description. Interestingly, models based on these representations have proved to better capture vehicular and pedestrian movement than regular models (Hillier and Iida, 2005). Such movement, as well as other kinds of human flows in urban space, is essential for urban modeling, since increasingly flows of this kind are understood as drivers in urban processes (e.g., Batty, 2013).

Critical for a full understanding of space syntax modeling is the ontology of its' representations, such as the axial map discussed below. The issue has given rise to several approaches in the space syntax literature, that not are immediately congruent. Through the years, there has been aims to view these representations from the point of view of phenomenological geography (e.g., Seamon, 2007), systems biology (Griffiths and Quick, 2005), neuroscience (e.g., Sakellaridi et al., 2015), or spatial cognition (Conroy Dalton et al., 2012). While this discussion remains inconclusive, there has also been a more technical debate, propelled by the rapid increase in accessible geodata on the internet (Stavroulaki et al., 2017). Essential here is the shift from Axial maps drawn by hand in GIS to maps based on Road Center Lines downloaded from, for instance, Open Street Map or different national road authorities. While this primarily has been driven by convenience, in that it is less time consuming to download a road system than to draw one yourself, and by incentives to adapt to other directions in urban modeling, where this is standard procedure, it again highlights the issue of the ontology of these representations. Moreover, there has been several proposals from within the space syntax field about how the axial map could be improved. One may identify four major directions here, (1) Angular Segment Analysis (e.g., Turner, 2007); (2) Natural Streets maps (Jiang and Claramunt, 2002); (3) Continuity maps (Figueiredo and Amorim, 2005); and (4) Directional Distance models (Peponis et al., 2008). These directions are thoroughly analyzed and compared in Stavroulaki et al. (2017).

Altogether, however, this leaves the status of the ontology of the representations in space syntax unsettled, where we by this simply mean; we do not quite know what they represent. To simply state that they are representations of space, or space as structured by built form, in the end leaves the issue far too open. Put differently, although we repeatedly see powerful correlations between analyses of the built environment based on space syntax representations and human behavior, especially movement, we do not have a theory that help us understand why this is so. This in turn hinders conscious improvements of the representations and ultimately also precision in the translations of these findings into policy and practice.

In this paper, we aim to remedy this by returning to the axial map, which we deem a highly original form of representation with a potentially strong foundation in psychological theory, in particular James Gibson's theory of affordances (1977), which we believe can shed light also on the ontological status of more recent development of the axial map, such as segment maps, as well as ready-mades, such as road center lines. Gibson's theory of affordances has often been referred to in the space syntax literature (e.g., Hanson, 2000), yet his broader theory about An Ecological Approach to Human Perception (1986), of which his affordance theory is part, has never been thoroughly discussed in relation to space syntax theory, despite the great affinity between the two theories on the matter of the humans-environment relation. Ultimately, we propose that Gibson offers a theory that help us understand why space syntax modeling works and that space syntax offers modeling techniques that make Gibson's theories operative on the scale of the city.

Hence, if this affinity can be substantiated into a distinct link, it may prove vital, both from the point of view of urban modeling, aiming to inform practices in urban planning and design, and environmental psychology, aiming to understand the relation between human behavior and the physical environment, and that in several respects. First, it opens for models that capture how human behavior is conditioned by the environment, which offers the opportunity to also model and understand how interventions of the built environment may influence human behavior. Second, it opens for models that capture how the same physical environment gives rise to different affordances depending on variations in the physical ability among humans, relating for instance to physical disabilities or age, but also, how the same physical environment gives rise to different affordances relating to other species than humans, such as birds or bees. Finally, it opens for combining affordances relating to both humans and other species in urban environments, which would substantially broaden the capacity of urban planning and design when it comes to transforming cities into greater social-ecological sustainability.

Hence, this paper aims to contribute to a stronger theoretical foundation of the form of representations developed in space syntax methodology, such as the axial map, by way of a close reading of Gibson's theories about affordances and ecological space, which also is intended as an argument for space syntax modeling as an approach that in contrast to much urban modeling bridges the humans-environment dichotomy. In extension, the aim is to also introduce to psychological theory a strand of urban modeling directly linked to important directions in psychology. We deem both important in the light of the shared aim of the two fields to create greater social-ecological sustainability in cities.

## Space syntax: an architectural approach to the humans-environment relation

The central components of any urban model are a set of modes to measure the key variables *distance* and *attraction* within a *representation of urban space* (Wilson, 2000). Distance can here be measured in many different ways, for instance as metric and temporal distance or as economic cost; attractions may be measured as anything from density of residents or accessibility to retail or parks. Similarly, urban space may be represented in many different manners, but the most common are either as coordinates in continuous space or as patterns of discrete spaces, for instance census tracts (Wilson, 2000). The issue of spatial representation becomes particularly interesting if we shift from a geographical to an architectural conception of space, since we in the latter find interesting alternatives, not least in what is known as space syntax research (Hillier and Hanson, 1984).

Space syntax origins in the 1970's debate about the need for sounder knowledge foundations for the design of new housing areas (Hillier et al., 1976). The background was the severe criticism of the ambitious British social housing program of the 1960's, where these, if anything, seemed to create social problems rather than remedy them. Similar criticism could be found throughout the western welfare states in the early 1970's. In most cases it embodied the same paradox: when the social ambitions had been the highest, architecture had failed the greatest.

An early contribution to this aim from the main originator of space syntax theory, Bill Hillier, concerned a critique of the established conception of the humans-environment relation (Hillier and Leaman, 1973). This line of argument was again taken up by Hillier in Space is the Machine (1996), which we here will follow closely in the aim to both identify the foundations of space syntax theory in this respect, but also in the aim to closer tie space syntax theory to Gibson's conception of embedded cognition.

Hillier follows Canguilhem (1971) in identifying the origin of the environment concept in emerging disciplines like biology and zoology in the seventeenth century (Hillier, 1996). The central issue here was to explain the great variety of species on Earth, where one, following the ideal of physics, looked for material explanations. The idea evolved that this was a result of the environment, typically conceptualized in a rather awkward mechanistic way that later often has been ridiculed. Hillier argues that this idea, even so, lived on into the twentieth century in architecture, translated into a paradigm of the machine; the idea that architecture through its' materiality somehow is able to influence, change and direct individual behavior. He in particular points out how a central problem here is how this idea reinforces the subject-object duality: “This blinds the inquirer to the most significant single fact about the built environment: that it is not simply a background to social behavior—it is itself a social behavior” (Hillier, 1996, p. 300). The reason it can be seen as such, according to Hillier, is that the built environment not is a natural environment, it is an artifact, and as such shaped according to basic human abilities with the very aim to condition and direct human behavior.

It is important to recognize the two-front character of the argument pursued here. At heart, space syntax springs from a critique of social engineering, architectural determinism and the paradigm of the machine, but it does not abandon either a belief in positive knowledge or a systematic relation between space and society, as much of the 1970's critique did. Hillier means that the latter critique led to a decisive change in architectural research from analytical studies of function, to hermeneutical studies of meaning, that is, an altogether different topic, somewhat mirroring the debate between behavioral and cognitive approaches in psychology (e.g., Sörqvist, 2016). In short, Hillier and Hanson want to save the idea that architecture has social effects, cognitively through the reading of architectural signs as well as behaviorally through the use of spatial form, by the means of a new and better scientific paradigm, where the critical difference from the earlier one is a shift from theory based on a direct relation between the physical environment and humans, to one where this relation is mediated by *spatial configurations*.

The central argument here is that human use of space must be understood dynamically, that is, through human movement in space rather than as static uses in particular spaces. This put emphasis on the relation between spaces, their configuration, rather than the physical form of individual spaces *per se*—we see the reason for the name space syntax. This, next, leads to the observation that in real life different human uses typically overlap in space rather than stick to particular spaces. Hillier and Hanson call this a *non-correspondence relation* between space and use, in contrast to the conventional *correspondence relation* between the two (Hanson and Hillier, 1987). This means that human use of space for movement has a vital and intermediate role in relation to other human uses or social phenomena in urban space. The configuration of space, as captured in the axial map, has then in a long line of empirical tests proven to an important degree structure movement patterns in urban space so that we find different numbers of co-present people in different urban spaces. This, in turn, create particular situations of varying social and economic potential in these spaces, relating to such things as social integration (e.g., Legeby, 2013) and local markets (e.g., Scoppa, 2015).

Importantly, such movement patterns are also essential when it comes to the encounter between people and urban green, central for changing people's behavior and attachment to environmental issues and improved sustainability (Marcus et al., 2016). This happens in three ways. First, in that the configuration of space structures movement so that it to greater or lesser extent pass urban green areas. Second, in that these movement patterns generate particular situations of co-presence in urban green areas that become part of their experience—whether there are 2, 20, or 200 people co-present around a water pond in a park dramatically changes its meaning. Third, in that depending on the size of these co-presences the landscape design may change to create more useful encounters between people and urban green. The two latter emphasize how space syntax methodology also can address symbolic and affective affordances and not only functional.

We may illustrate this with an example. Imagine a city where everyone walks to work each morning, whether these people encounter any form of urban green on their journeys, naturally depends on the distribution of green areas in the city but also on the configuration of the street network. Walking to work normally implies simple and direct journeys, which according to the arguments above, generally means streets of high centrality in the street system, hence we need to distribute green areas along these particular streets for such encounter to happen. In the next step, however, this also means that green areas in these locations will be frequently visited, that is, I normally will encounter the green together with others and often with many others. This creates a particular situation for the encounter with the green; it is somehow “colored” by the large group of others. This naturally does not mean that this is bad, but it is different from a situation where you encounter the same green alone or together with few other people.

The first case may also imply that one should design the urban green in a particular way to accommodate for this particular situation of quite few simultaneous visitors. For instance, large groups of people mean a lot of wear and tear, why areas covered with grass need certain treatment or perhaps even to be avoided, not to present nature as something worn and dirty to visitors, which then may come to prefer shopping malls with flower pots. In contrast, more segregated space may be designed in a very different manner and also have a different function for human interaction with nature. There naturally are many dimensions to this example, but in principle it demonstrates how movement patterns and the situations of co-presence it generates is essential for building a relation between humans and nature in cities, where the point in our current discussion is that we can learn to better model and understand these patterns through space syntax models based on representations of certain human affordances.

Naturally, every step in this argument, while empirically supported in many studies (e.g., Hillier and Iida, 2005), has also been debated: from the representation of the axial map itself (Batty, 2013), over to its distance measure (e.g., Jiang and Claramunt, 2002), its statistical correlation with number of co-present people (e.g., Ståhle et al., 2005), and the social interpretation of these situations of co-presence (Liebst, 2014). In relation to current urban development challenges, it also demonstrates limitations, where major directions in need of development concern, joining the axial map with other models of urban mobility (Gil, 2016), incorporating also urban green structures (Berghauser Pont et al., 2017), and extending the models over time, allowing for dynamic analysis.

Hence, space syntax is a field still in development but has introduced a novel and substantiated approach to the humans-environment relation by way of spatial configuration: “Spatial configuration proposes a theory in which we find pattern effects from space to people and from people to space that in no way invokes mechanistic determinism. At the same time, the configuration paradigm saves the idea that architecture has social effects. By changing the design of a building or complex we do change outcomes. There is after all some kind of mechanism between the built world and people. However, the machine is not the building. *Space is the machine*” (Hillier, 1996., p. 300).

## Space syntax: cities as cognitive objects

To understand how space may become a machine, we need to better understand properties of space in relation to basic human capacities and how humans by making use of these properties, have rearranged the environment to their own purposes. Hillier maintains that people interact with space in cities both through their capacity as bodies and as minds and argues that: “in bodily terms the city exist for us as a system of *metric distances*,” while we mentally interact with it primarily by seeing: “as a system of *visual distances*” (Hillier, 2012, p. 4). We here see the theoretical foundations for the axial line, which simply is a geometric representation of an urban space structured and limited by built form, that is possible to physically access and visually overlook for a generic human.

However, Hillier also argues that: “we also need to reflect on the fact that cities are also collective artifacts which bring together and relate very large collections of people. The critical spatial properties of cities are not then just the relation of one part to another, but of *all parts to all others*” (Hillier, 2012, p. 4). Here we see the need to expand the axial line to an axial map, which is a set of axial lines covering all spaces accessible for a generic human within a spatial system structured and limited by built form. As a means to measure distances within such a system, Hillier next proposes the notion of *universal distance* as opposed to *specific distance* (Hillier, 1996, p. 104–108). Where specific distance concerns the distance between an origin A and a destination B, universal distance concerns the distance from all possible origins to all possible destinations in a spatial system. Hence, universal distance comes close to the concept *centrality*, for instance found in network analysis (Newman, 2010).

**Figure 1:**
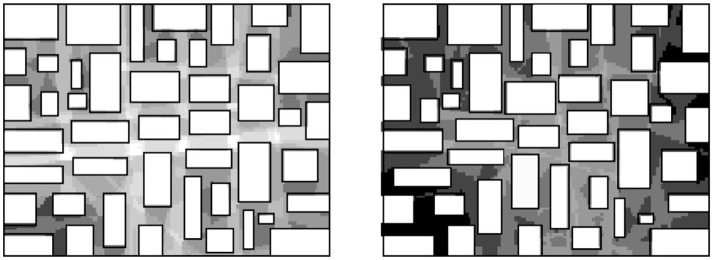
Analysis of universal visual distance in a grid-like bounded space, where small displacements of the blocks cause dramatic increase in universal visual distance (Hillier, 2012).

According to Hillier, universal distance behaves differently from regular ideas about distance (Hillier, 2012, p. 5). He illustrates by filling a bounded space with a set of rectangular objects, resembling urban blocks, in such a way that the remaining space takes a form that looks like a street grid (Figure 1). By moving the blocks slightly, so that some of the lines of sight are broken, Hillier shows how visual distances are increased dramatically, while the metric distances changes only marginally. This reflects the common experience that the same metric distance can be experienced very differently depending on the particular spatial situation; for instance, one can imagine how trips in the second system will both take more time as well as represent a greater effort than in the first system; that is, not a much greater physical effort but a much greater mental effort.

The argument for the axial line as a distance unit can then be made: If we make a straight line crooked “we do not add significantly to the energy effort required to move along it, but we do add greatly to the informational effort required” (Hillier, 2003, p. 3). Based on the idea that cities, as products of bottom-up incremental processes over long time (see Alexander, 1964), have tended to evolve toward environments that support human intelligibility, Hillier concludes that: “human geometric intuitions seem to be *embedded* in the city itself” and in extension that: “cities are in a profound sense *cognitive*—and so human—objects before they are economic and social objects” (Hillier, 2012, p. 18). We may then, at least in part, also identify a possible explanation to the failure of the large-sale housing programs of the 1960's, which typically did not evolve in relation to human practice over large time spans, but rather were rapid large-scale interventions, little informed about human cognition or use of the environment.

**Figure 2:**
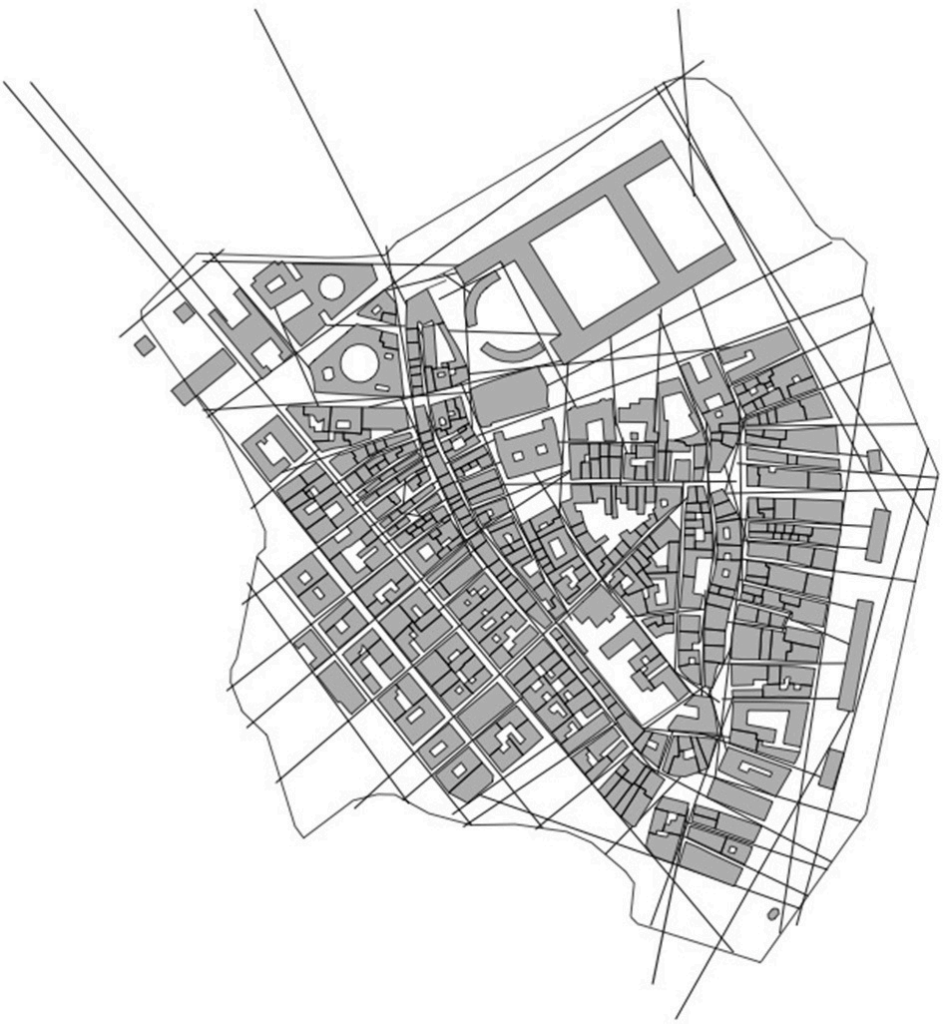
The axial map as a representation of spatial.

On this basis, we can begin to more precisely understand what the axial map represents (Figure 2). It is a representation of continuous urban space, structured by built form such as buildings, infrastructure and landscape elements, which specifically captures a vital set of affordances (accessibility and visibility) that the environment gives rise to in relation to a moving human subject. It does so by representing urban space as the least amount of straight (axial) lines that completely covers the whole system under analysis. Hence, the axial map can be interpreted as an attempt, with the simplest geometry possible, to represent this set of affordances. Finally, each axial line can then be used as a unit for topological measurement of distances, which can be argued to be based in human perception and cognition.

We here see the essential importance in choice of geometric representation in urban modeling, where the axial line in this case, despite its' mundane appearance, on the one hand, constitutes a kind of *cognitive geometry*, in that it represents the spatial environment (the object) from the point of view of a perceiving and cognizing human (the subject), that is, a representation that neither is a representation of the subject or the object, but rather a representation that overcomes the categorical separation of the two. On the other hand, this representation opens for precise description, analysis, and even quantification of this geometrically constructed entity. For instance, we may topologically measure the number of axial lines between two locations or, in the same manner, measure the centrality of a location in a spatial system, which we in both cases may call an analysis of *cognitive distance*. It is this manner of measuring distance, that is, as topological steps of axial lines in an urban environment represented as an axial map, that in repeated empirical investigations has proven to capture both pedestrian and vehicular movement better than other distance measures (e.g., Hillier and Iida, 2005).

## Space syntax: a copernican shift in urban network analysis

**Figure 3:**
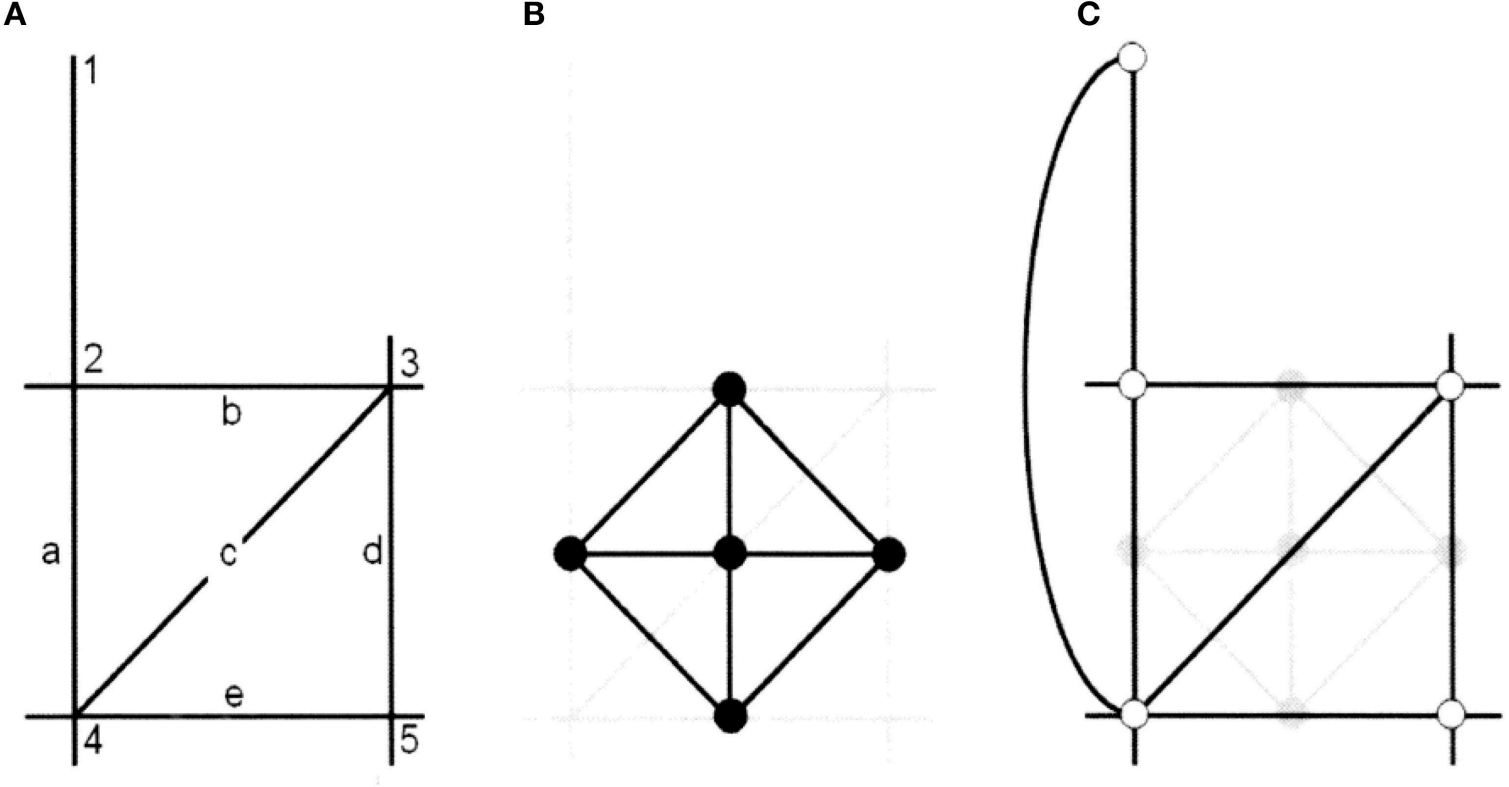
The Copernican shift in graph representations of urban space in space syntax (Batty 2013). **(A)** The street network as an axial map. **(B)** The primal syntax between streets/lines. **(C)** The dual syntax between junctions/points.

When we come to analysis, axial maps and later developments into segment maps etc. (see note 1), are all treated as networks, and as such formally described by graphs and thereby part of a long tradition of applying graph theory in spatial analysis and urban modeling (Batty, 2013). A common procedure here is to represent urban elements, such as buildings, parks, or retail, as nodes and the relations between these as links in a network matrix (Batty, 2013). When embedding such a network in a real urban setting, however, one most often make use of the street network for setting urban elements in spatial relations to each other. When representing such street networks as graphs one normally represents street-junctions as nodes and the street-segments connecting these as links. Peculiar to the axial map, however, is that this is done the other way around; streets are represented as nodes and junctions as links. In formal graph terms, there is always such a *dual graph* to a network, the mirror image of the *primal graph* (Figure 3), and it is this dual graph that is made use of in space syntax.

This procedure is logical to the conception of space we find in space syntax, since it means that we represent urban space as a set of spaces defined by a certain set of affordances (accessibility and visibility), where each such space is represented as a node in the graph and each junction of such spaces is represented as a link. Representing the axial lines as nodes and the junctions between axial lines as links, rather than the opposite, means that the cognizing subject, so to speak, is shifted from the street-junctions to the street-segments and, moreover, from being represented as a point to being represented as a line. We propose that this has far greater consequences than what it seems and actually constitutes something of a Copernican shift in urban network modeling.

This is especially true, since what is represented by the axial lines, as we have seen, are spaces possible for a human to physically access and visually overlook, and such spaces are normally not limited to the space of a street segment, but regularly extend beyond such segments, while at times also being shorter than a segment. This has the rather contradictory effect that an axial line can be connected to several other axial lines, while these lines, in turn, not are connected to each other. In relation to human perception, however, this makes sense; even though we in a physical sense are located at a specific point, we may visually overlook a much larger area, potentially including several street-junctions, and in that visual sense be present also at these other street-junctions—the axial line in an original manner captures both of these dimensions.

Hence, we see a different conception of space in space syntax compared to regular urban modeling, a conception, we will argue, comes very close to what Gibson has called an ecological conception of space in contrast to the conception of space in physics, which is what we normally encounter in urban modeling.

## Ecological space: entering the world of meaningful things

Gibson's particular point of departure is well-captured by the title of his book: An Ecological Approach to Visual Perception (1986). The term “ecological” marks a break with physicist conceptions of the environment and point to our habit to speak about reality as the physical world and, as a consequence, the risk of uncritically adopting physicist conceptions of the environment. In contrast to such conceptions he states the rather obvious fact that from the point of view of psychology: “we are concerned here with things at the ecological level, with the habitat of animals and men” (Gibson, 1986, p. 9), which concerning our understanding of space constitutes a subsection of physics, albeit a subsection we need to understand better.

In Gibson's own words, his theory “provides a counterbalance to those theories of cognitive mapping that have focused mainly or only on the internal cognitive processing of environmental information, to the exclusion of any interaction of the individual and the environment” (Gibson, 1986, p. 13). More precisely, he wants to open for an understanding of the body as an extension of the mind, together constituting an integrated perceptual system, rather than a sequence where the one follows upon the other. It also explains Gibson's unwillingness to make a distinction between perception and cognition. He argues: “Our reasons for supposing that seeing something is quite unlike knowing something come from the old doctrine that seeing something is having temporary sensations one after another at the passing moment of present time, whereas knowing is having permanent concepts stored in the memory” (Gibson, 1986 p. 258).

Consequently, Gibson constructs a new ontology, based in an ecological conception of the world rather than a physical one. To give an example of what the difference here implies, we can compare the isotropic conception of space in physics, defined by an x, y, and z-axis, with ecological space conceptualized as what surrounds living organisms, where the latter immediately need to acknowledge the primacy of the ground, for instance; for an experiencing human, there simply is no spatial isotropism, the way physics tend to deal with space. In Gibson's words: “The world of physical reality does not consist of meaningful things. The world of ecological reality, as I have been trying to describe it, does” (Gibson, 1986 p. 33).

First of all, Gibson instigates the mutuality of *animals*, which includes humans, and *the environment*, the fact that: “each term implies the other” (Gibson, 1986 p. 8). This conception of mutuality between animal an environment gives rise to the basic elements of his ontology: *medium, substances* and the *surfaces* that separate them, where the typical “media” are air and water, which allow animal movement, while the earth, and other hard materials that do not allow such movement, are “substances.” Interfaces between these, whether between different media or different substances or between a medium and a substance, all constitutes “surfaces” (Gibson, 1986 p. 16). The latter plays a critical role for perception in that they give structure to the light that surrounds us and thus allows for vision. Gibson calls this *ambient light*, which maintains its particular property from the fact that it concerns light in an environment, which causes rays of light, even though we assume a primary source of light such as the sun, to continuously reflect so that we can think of them as coming from every direction, thus filling the medium. However, this also implies that an environment, constituted by a particular configuration of surfaces, typically will structure the ambient light in a certain way and give rise to what Gibson calls an *ambient optic array* that, in principle, is unique for every location (Gibson, 1986 p. 51). It is this structured array of light that enables light to carry information that can specify the environment for a perceiving animal, that is, that light from a point of observation simply will have different forms in different directions.

Of specific importance to architecture and urban design is Gibson's discussion about how the structure and shape of the environment creates what he famously has called *affordances* (Gibson, 1986 pp. 127–143). This concerns how a given environment affords, that is, presents certain potentials for behavior depending on the constitution of the bodies of different animals: “The *affordances* of the environment are what it *offers* the animal, what it *provides* or *furnishes*, either for good or for ill” (Gibson, 1986 p. 127). While we primarily may think of affordances as given by the natural environment it is obvious that many species, not only humans, invest a lot of energy and resources into transforming the environment according to their purposes, that is, they transform the environment to increase its affordance in relation to the needs of their own species. As Gibson notes (Gibson, 1986 p. 37), this can also concern the creation of obstacles in the environment, a form of “disaffordance,” to protect from or exclude other species. When it comes to the human species, such investments in affordances have taken on tremendous proportions transforming large parts of the Earth's surface.

Gibson's shift toward a conception of the mind and the body as an integrated perceptual system also implies that movement is essential to any kind of perception; from the movement of the eye balls in the head, over to the movement of the head at the top of the neck, to the movement of the body through the environment by means of walking: “Vision is a whole perceptual system”; in short, even though: “one surely *looks* with the eyes […] one does not *see* with the eyes” (Gibson, 1986 p. 205). With the idea of the body as a perceptual system, Gibson makes a decisive break with conventional cognitive science, especially as developed in neuro science, where the body and the head in many experiments, at least traditionally, were forced to keep still in the aim to investigate the brains reaction to different controlled stimuli. His point here is that these experiments may be useful if we want to understand how the brain works, but they are not realistic if we want to understand how humans perceive their environment. In the latter case movement is essential, and in the end what distinguishes animals from other organisms is that they can move—it is their competitive advantage.

Human movement, especially bodily locomotion, is what sets Gibson's ontology into action, so to speak. The medium affords human locomotion but is structured by substances. The substances, however, are not mere obstacles but offer permanence against which movement can be sensed and controlled. More specifically this happens through the changing configuration of surfaces that continuously come into and move out of the field of vision, hence structuring and restructuring the ambient light which thereby carries information about the environment to the perceiving human. From the information point of view the tension between change through human locomotion and permanence offered by the environment is critical: “The optic array changes, of course, as the point of observation moves. But it also does *not* change, not completely. Some features of the array do not persist and some do. The changes come from the locomotion, and the non-changes come from the rigid layout of the environmental surfaces. Hence, the non-changes specify the layout and count as information about it; the changes specify locomotion and count as another kind of information, about the locomotion itself” (Gibson, 1986 p. 73). As a matter of fact, all individual perception is in this sense continuous, why each moment of perception, in principle, happens against the background of earlier moments of perception, why these are part of the present moment of perception. We simply cannot exclude memory from perception and, therefore, the distinct divide between perception and cognition proves difficult to sustain, according to Gibson.

If we acknowledge the fact that humans primarily perceive the environment under movement and that arrested vision rather is the limiting case, this proves to have quite radical implications. First, as we have seen, that the past somehow is present in the present, so to speak; what we just saw help us perceive what we see now, that is, that we cannot discount memory in perception. While this manner of understanding perception, hence, dislocates the *moment* of observation, as it were, it similarly, second, also dislocates the *location* of observation: “Seeing the world at a traveling point of observation, over a long enough time for a sufficiently extended set of paths, begins to be perceiving the world at *all* points of observation, as if one could be everywhere at once” (Gibson, 1986 p. 197). While this at first seems to be an odd conclusion, upon reflection it seems to be a rather apposite description. What we perceive when we move around the environment cannot really be said to be a series of images of this environment, but rather a complete conception of that environment, however imperfect, where certain parts stand out more than others, that is, closer to a 3D-model.

## Cognitive geometry: representing human-environment relations as things

We clearly see connections between the two conceptions of the relation between humans and the environment in the two discussions above; space syntax looking for powerful representations that capture humans embedded in the environment and Gibson identifying affordances as an immediate link between humans and the environment. Most interestingly in this respect, Gibson states: “An observer who is getting around in the course of daily life sees from what I will call a *path* of observation” and, furthermore: “the medium can be thought of as composed not so much of points as of paths” (Gibson, 1986 p. 197). If we earlier have seen how what Gibson calls the medium, in an urban setting is structured by particular configurations of surfaces into spatial form, we can see how what Gibson is saying here is that such spatial form, can be represented by a line, or, potentially, a set of lines.

Gibson develops this idea in the context of animal orientation, which he grounds in what he calls the theory of *reversible occlusion*. He describes this in great detail, which proves very supportive for our attempt to link his ideas to space syntax representations, such as the axial map:

“An alley in a maze, a room in a house, a street in a town and a valley in a countryside each constitutes a place, and a place often constitutes a vista, a semienclosure, a set of unhidden surfaces. A vista is what is seen from here, with the proviso that ‘here’ is not a point but an extended region. Vistas are serially connected since at the end of an alley the next alley open up […]. To go from one place to another involves the opening up of the vista ahead and closing in of the vista behind […] When the vistas have been put in order by exploratory locomotion, the invariant structure of the house, the town, or the whole habitat will be apprehended. […] It is not so much having a bird's-eye view of the terrain as it is being everywhere at once”. (Gibson, 1986 p. 198).

It is this perception under movement and the sense of the environment it generates that he means explains the capability of orientation: “To the extent that one has moved from place to place, from vista to vista, one can stand still in one place and see where one is, which means where one is relative to where one might be” (Gibson, 1986 p. 200). Since what we do, according to Gibson's argument, is to continuously scan the environment rather than take snapshots of it, we are in the end able to generate a, more or less, shared perception of the world.

**Figure 4:**
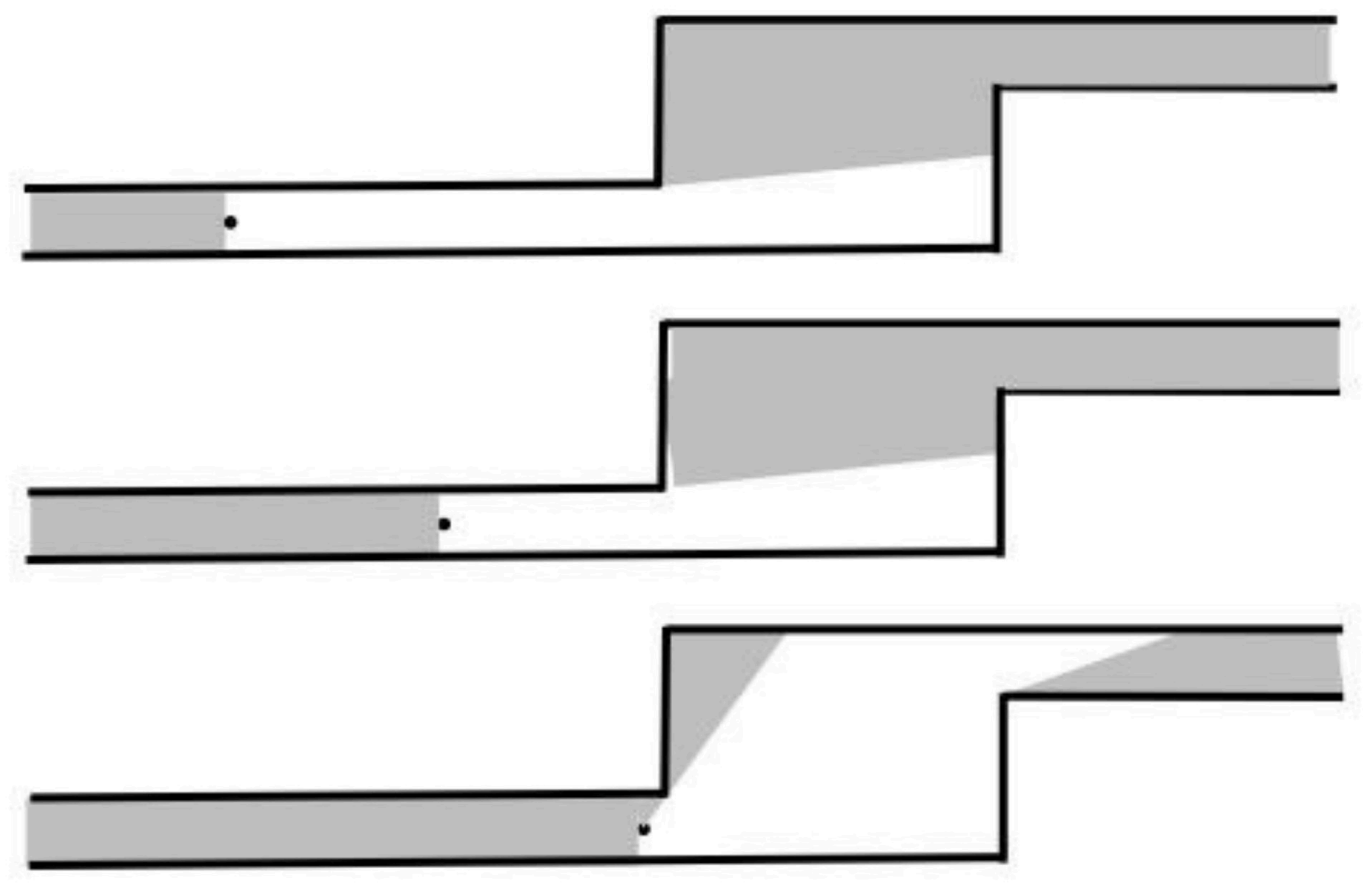
The opening up of a vista at an occluding edge, as seen from above (After Gibson, 1986).

Upon closer examination, the axial map turns out to be something that comes very close to Gibson's description above (Figure 2); a network representation of spatial form from the point of view of what we may call a cognitive subject, that is, a perceiving human being moving through space. This is almost an identical description to the one we find when Gibson attempts to illustrate his theory of reversible occlusion (Figure 4) (Gibson, 1986, p. 199). In the figure, we see how the “perceptual spatial unit” continuously changes as the observer moves through space (the medium), due to the physical structure of built form (the substance), that is, particular configurations of built form (surfaces) come into and goes out of sight for the observer, creating a continuous set of vistas. This captures Gibson's idea that we do not perceive a sequence of discrete vistas when moving through the environment, but rather a spatial continuum where large parts of the environment typically remain invariable so that what we develop is a conception of the environment as perceived from everywhere.

Now, what we find in the axial map is not a representation of such a spatial continuum but the least amount of “perceptual spatial units” that cover the area we want to represent (Figure 2). These units are represented as lines distinctly crossing each other as to both connect to each other as a form of continuous path and to not leave any space possible to access and perceive outside the representation. The axial map thus constitutes a kind of representation, of great economy, of what an urban environment *affords* the visibility and accessibility of a generic human. The representation of this continuous medium in the form of the least possible amount of “spatial units” of course represents a reduction of reality, typical for any modeling, but the gain is that the continuous medium of space, which is highly difficult to analyse as such, is transformed into a distinct set of elements possible to represent as a network and also to analyse as such.

Informed by this discussion we may now return to the ontological nature of the axial map. We may ask: is it a representation of the human subject's conception of urban space or is it a representation of the environmental object's spatial reality. We propose, that it is neither, or rather both, that is, a representation of urban space that by starting in the perception of a human moving in such space, captures what the environment affords its perception. Hence, affordances are neither part of the environment or of humans, but rather belong to a human-environment system. The axial map is then not only a representation of a spatial network where the individual lines represent different spatial units tied together at their crossings, but rather a network of affordances, that is, a series of human-environment relations, where each line in itself represents such a relation between humans and the environment; hence the human subject is written into the axial map to the same degree as the physical environment.

## Conclusions: linking psychology to the urban planning and design of sustainable cities

From the discussion above we can draw some important conclusions. From the point of view of space syntax, we, first, see that, in contrast to most representations of urban space, the axial map deals with ecological space in Gibson's meaning of the word, rather than physical space. Second, what is represented by the axial map is not space but the situated affordances that emerge in the encounter between human abilities and environmental features. This means, third, that we have a theory about what it is in these representations that has proven so powerful in empirical studies of human movement, which in turn could form the basis for precise empirical tests of this theory. Fourth, this also opens for the possibility that developments of the axial map, such as the segment map or continuity line maps, also can be scrutinized and tested as representations of human affordances, where they may be found as improvements of the axial map in this regard.

From the point of view of psychology, we may conclude, first, that space syntax, and especially the axial map, offers a unique link between psychological theory and urban modeling, which in turn means a link between psychology and the practices in urban planning and design. Second, we see how space syntax can be useful both in relation to behaviorist and cognitive approaches in psychology to increased sustainable behavior. On the one hand, we have seen how it has proven successful in capturing, what we may call, the behavioral substratum for sustainable behavior in a more direct sense, in the form of movement patterns and the distribution of co-presences in urban space. But also, on the other hand, how these situations of co-presence create the foundations for specific design of human encounter with urban green, where particular cognitive experiences relating to values, beliefs, and norms can be generated.

Hence, we see a highly interesting link between a field concerned with understanding the relation between humans and their environment and a field that generate knowledge about how to intervene and change urban environments, at a time when we urgently need to redirect our cities into more sustainable trajectories.

## Author contributions

The author confirms being the sole contributor of this work and approved it for publication.

### Conflict of interest statement

The author declares that the research was conducted in the absence of any commercial or financial relationships that could be construed as a potential conflict of interest.

## Footnotes

^1^We will only refer to the axial map here, while keeping in mind the general implications of our argument for other geometric representation typical for space syntax, such as the segment map or the isovist. For full review of these representations, see Stavroulaki et al. (2017).
